# The relationship between organisational support for career development, organisational commitment, and turnover intentions among healthcare workers in township hospitals of Henan, China

**DOI:** 10.1186/s12875-022-01753-4

**Published:** 2022-06-02

**Authors:** Huan Wu, Yaqing Liu

**Affiliations:** 1 School of Medicine and Health Management, Tongji Medical College, Huazhong University of Science and Technology, Wuhan, Hubei China; 2 School of Management, Xinxiang Medical University, Xinxiang, Henan China

**Keywords:** Organisational support for career development, Organisational commitment, Turnover intention, Healthcare workers, Township hospitals

## Abstract

**Background:**

Township hospitals in China face the challenge of shortage and turnover of healthcare workers. This study aims to evaluate the relationship between organisational support for career development (OSCD), organisational commitment, and turnover intentions among healthcare workers in township hospitals.

**Methods:**

The data in this study were obtained from the Health Service Capacity Survey of Primary Health Institutions (2020), one of the special surveys of rural health poverty alleviation in Henan, China. The sample comprised 298 healthcare workers. Three standardised instruments were used: the turnover intention scale, OSCD scale, and organisational commitment scale. Descriptive statistics, One-way Analysis of Variance, Pearson correlation analysis, and the simple mediation model were used for data analysis.

**Results:**

The results showed that the mean score of the healthcare workers’ turnover intentions was 2.21 ± 0.77, which was low. The healthcare workers’ turnover intentions had significant differences in gender, age, marital status, education, professional title, and organisational tenure. OSCD had no significant direct relationship with turnover intentions, while having a significant positive direct relationship with organisational commitment. Organisational commitment had a significant negative direct relationship with turnover intentions, and played a fully mediating role in the relationship between OSCD and turnover intentions.

**Conclusions:**

OSCD had an indirect negative effect on healthcare workers’ turnover intentions in township hospitals through the full mediation of organisational commitment. The findings of this study suggest the importance of policymakers and organisation managers to improve OSCD practice and pay attention to ‘humanistic management’. In addition, the formulation and implementation of talent retention policies should consider socio-demographic differences.

**Supplementary Information:**

The online version contains supplementary material available at 10.1186/s12875-022-01753-4.

## Introduction

Healthcare workers are core elements constituting the service capacity of health institutions and an important guarantee for health institutions to perform their functions effectively. In practice, however, township hospitals in China face the challenges of a shortage and turnover of healthcare workers. A previous study has reported that the average brain drain rate of township hospitals was 3.45%, of which approximately 80% were healthcare workers [1]. Turnover among healthcare workers leads to a decrease in the efficiency of health service supply, hindering rural residents' access to quality and safe health services [2, 3]. Therefore, it is worth exploring ways to reduce the turnover rate of healthcare workers in township hospitals in China.

The turnover intention is the process in which employees think of quitting their current jobs and plan to find new opportunities [4]. It is considered one of the best factors to predict employee turnover behaviour [5]. Therefore, the evaluation of the turnover intention can help prevent turnover behaviour. During the past few decades, there has been no shortage of studies on the brain drain of township hospitals in China. These studies have examined many strong predictors of healthcare workers’ turnover intentions, such as job satisfaction, work stress, occupational stress, and burnout [5–9].

Organisational support for career development (OSCD) is a programme or process by an organisation for the career development of its employees, and it is also called organisational career management policy and practice, or organisational career management [10]. As an organisational resource, some studies have shown that the OSCD is negatively related to turnover intention [10, 11]. However, to the best of our knowledge, a few studies have focused on the relationship between OSCD and turnover intentions among healthcare workers in township hospitals. Moreover, these studies only analyzed the direct effect of a specific OSCD practice on turnover intention, and did not explore the potential of OSCD as a system [6, 12].

Organisational commitment is the strength of an individual’s identification with and involvement in an organisation [13]. As a positive emotional experience, many previous studies have consistently found that organisational commitment can reduce employees’ turnover intentions [13, 14]. Furthermore, a previous meta-analysis showed that perceived organisational support was negatively related to organisational commitment [15]. It is inferred that there may be a relationship between OSCD, organisational commitment, and turnover intentions. However, in health and other areas, research on the relationship between OSCD, organisational commitment, and turnover intentions is limited.

Considering this, based on a cross-sectional survey of healthcare workers in township hospitals in Henan Province, this study aims to evaluate the relationship between OSCD, organisational commitment, and turnover intention. It enriches the literature on the brain drain of township hospitals, providing valuable information for human resource management and reform of township hospitals.

## Theoretical background and hypotheses

The social exchange theory refers to the basic psychological process of people in social exchange and its relationship with exchange behaviour. The theory holds that individuals in interpersonal relationships are motivated by the positive outcomes they expect [16]. The social exchange theory has been widely used in research on organisational support [17, 18]. When employees perceive the organisation’s support for them, their psychological contract with the organisation will be strengthened, reducing their turnover intentions [10]. Based on this theory and previous empirical evidence, we formulated the following hypotheses:

- Hypothesis 1: OSCD is negatively related to healthcare workers’ turnover intentions.
- Hypothesis 2: OSCD is positively related to healthcare workers’ organisational commitment.
- Hypothesis 3: Organisational commitment is negatively related to healthcare workers’ turnover intentions.
- Hypothesis 4: Organisational commitment mediates the relationship between OSCD and turnover intentions.

Based on our hypotheses, OSCD will have a direct effect on turnover intentions; furthermore, it will also have an indirect effect on turnover intentions through organisational commitment.

## Methods

### Study population and sampling

The data in this study were obtained from the Health Service Capacity Survey of Primary Health Institutions (2020), one of the special surveys of rural health poverty alleviation in Henan, China. The survey covered health institutions and healthcare workers. The healthcare workers included healthcare service providers, such as physicians, nurses, pharmacists, and medical laboratory workers. Administrators and rear service personnel were excluded from the study. This survey was conducted using stratified random sampling. First, the province was divided into three regions according to its level of economic development (high, medium, and low). Second, two counties in each region were randomly selected, resulting in a total of six. Third, five township hospitals were randomly selected from each county, resulting in a total of 30 such hospitals. Next, 10–12 healthcare workers were randomly selected from each township hospital, resulting in a total of 346 healthcare workers. Participants who gave written informed consent and agreed to participate in the survey were scheduled to complete the questionnaires independently. A total of 48 questionnaires were rejected because of incomplete or suspected incorrect answers. Finally, 298 eligible questionnaires remained, with an effective response rate of 86.13%. This survey was approved by the Ethics Committee of the Xinxiang Medical University (reference number XYLL—2,017,032).

### Data collection instruments

The questionnaire items related to this study included the following four sections: socio-demographics, OSCD, organisational commitment, and turnover intentions. The socio-demographic variables were as follows: (1) gender; (2) age; (3) marital status; (4) education; (5) profession; (6) professional title; (7) organisational tenure; (8) average monthly income; (9) hours worked per week.

Scholars have reached a consensus on the multi-dimensional nature of OSCD [19–21]. In the Health Service Capacity Survey of Primary Health Institutions (2020), the OSCD was measured using a 10-item scale developed by Sturges et al. [21]. The scale reflected two dimensions of the OSCD, namely formal and informal. The formal OSCD was assessed using six items. Some items were: ‘I have received training to help develop my career’ and ‘I have been offered a personal development plan’. The informal OSCD was assessed using four items. Some items were: ‘I have been arranged a mentor to help me with my career development’ and ‘I have been given fair career development advice from my organisation when I need it’. Respondents answered on a 5-point Likert scale ranging from 1 (strongly disagree) to 5 (strongly agree). In this study, the scale reported excellent reliability. The Cronbach’s alpha value of formal and informal OSCD was 0.90 and 0.93, respectively, and that of all the items on an average was 0.95.

Organisational commitment was assessed using a 9-item scale developed by Mowday [22]. Some items were: ‘I care deeply about the fate of this organisation’ and ‘I am willing to accept almost any kind of work arrangement as long as I can stay in this organisation’. Respondents answered on a 7-point Likert scale ranging from 1 (strongly disagree) to 7 (strongly agree). Cronbach’s alpha for the present study was 0.95, and the scale had excellent reliability.

The turnover intention of healthcare workers was measured using a 6-item scale [23]. Some items were: ‘Maybe I will not stay with this organisation for much longer’ and ‘I often think about quitting’. All items were measured on a 5-point Likert scale ranging from 1 (strongly disagree) to 5 (strongly agree). In this study, Cronbach’s alpha value was 0.85, indicating acceptable reliability.

### Statistical analysis

Descriptive statistics were used to describe participant characteristics. Specifically, frequencies and percentages were used for categorical variables, while means and standard deviations (SD) were used for continuous variables. One-way Analysis of Variance (ANOVA) was used to assess the differences in turnover intention scores of healthcare workers in terms of socio-demographic characteristics. Pearson correlation analysis was used to measure the correlations among study variables. The simple mediation model (model number: 4) in SPSS (V25) PROCESS macro (4.0) was used to test the hypothetical relationships of the proposed factors [24]. To ensure the accuracy of the mediation effect inference, the bootstrap 5000 sampling technique was used in the simple mediation model. To mitigate confounding bias, socio-demographic variables with significant differences in turnover intentions were added to the model as control variables.

## Results

### Socio-demographic characteristics

The respondents’ socio-demographic characteristics are presented in Table 1. Among the 298 healthcare workers, 43.62% were men, and 71.14% were under 40 years of age. The largest proportion of respondents (81.88%) was married. In terms of education levels, respondents who had junior college degrees accounted for 40.27%, which was higher than those with other degrees. More than half of the respondents were doctors (51.01%) and nearly 20% were nurses (19.46%). Furthermore, 38.26% and 30.87% of respondents held junior and middle professional titles respectively. Approximately 27% of healthcare workers had worked in their current health facilities for less than five years. Most (75.84%) of the surveyed healthcare workers had an average monthly income of less than 4,000 RMB Yuan. Additionally, 77.18% worked for more than 40 h per week.

**Table 1:** Socio-demographic characteristics of healthcare workers (*N* = 298)

Characteristics	*N* (*%*)	Turnover intention (*Mean* ± *SD*)	*F*	*p*-value
Gender				
Male	130(43.62)	2.11 ± 0.75	4.42	0.04
Female	168(56.38)	2.29 ± 0.78		
Age				
< 30	72(24.16)	2.53 ± 0.87	10.18	< 0.01
30 ~	140(46.98)	2.18 ± 0.67		
40 ~	86(28.86)	2.00 ± 0.77		
Marital status				
Unmarried	54(18.12)	2.58 ± 0.77	15.80	< 0.01
Married	244(81.88)	2.13 ± 0.75		
Education				
High school/Technical school or below	72(24.16)	1.97 ± 0.60	7.60	< 0.01
Junior college	120(40.27)	2.18 ± 0.77		
Bachelor or higher	106(35.57)	2.42 ± 0.83		
Profession				
Physicians	152(51.01)	2.25 ± 0.84	0.44	0.78
Nurses	58(19.46)	2.16 ± 0.56		
Pharmacists	18(6.04)	2.04 ± 0.50		
Medical laboratory workers	16(5.37)	2.25 ± 0.82		
Other healthcare workers	54(18.12)	2.20 ± 0.84		
Professional title				
No	60(20.13)	2.44 ± 0.89	3.08	0.03
Junior	114(38.26)	2.24 ± 0.71		
Middle	92(30.87)	2.07 ± 0.67		
Senior	32(10.74)	2.09 ± 0.92		
Organizational tenure				
< 5	80(26.85)	2.36 ± 0.90	3.63	0.02
~ 9	68(22.82)	2.25 ± 0.75		
~ 19	90(30.20)	2.24 ± 0.66		
20 ~	60(20.13)	1.94 ± 0.72		
Average monthly income (RMB Yuan)				
< 3000	124(41.61)	2.18 ± 0.81	1.01	0.37
3000 ~	102(34.23)	2.30 ± 0.73		
4000 ~	72(24.16)	2.14 ± 0.77		
Hours worked per week				
≤ 40	68(22.82)	2.27 ± 0.79	2.28	0.10
41 ~ 56	142(47.65)	2.27 ± 0.79		
57 ~	88(29.53)	2.06 ± 0.72		

### Turnover intentions of healthcare workers

The differences in turnover intention scores of healthcare workers in terms of socio-demographic characteristics are shown in Table 1. The OSCD (formal and informal OSCD), organisational commitment, and turnover intention scores and their correlations are shown in Table 2. As can be seen, the average score of the turnover intention was 2.21 ± 0.77, those of formal and informal OSCD were 3.79 ± 0.70 and 3.77 ± 0.87, respectively, and that of organisational commitment was 5.34 ± 1.24. Both formal and informal OSCD were positively correlated with organisational commitment (*r* = 0.69, *p* < 0.01; *r* = 0.74, *p* < 0.01). Formal and informal OSCD and organisational commitment were negatively correlated with turnover intentions (*r* = -0.29, *p* < 0.01; *r* = -0.31, *p* < 0.01; *r* = -0.41, *p* < 0.01). The ANOVA test showed that healthcare workers’ turnover intentions had significant differences according to gender, age, marital status, education, professional title, and organisational tenure. Specifically, higher turnover intention scores were presented in females (*F* = 4.42, *p* = 0.04), people less than 30 years of age (*F* = 10.18, *p* < 0.01), unmarried people (*F* = 15.80, *p* < 0.01), people with bachelor’s degrees or above (*F* = 7.60, *p* < 0.01), people without professional titles (*F* = 3.08, *p* = 0.03), and people working for less than five years in the current health facility (*F* = 3.63, *p* = 0.02). The differences of OSCD and organisational commitment scores in socio-demographic characteristics are presented in the supplementary file.

**Table 2:** Means, Standard Deviations, and Correlations of study variables (*N* = 298)

variables	*Mean*	*SD*	1	2	3	4
1. Formal OSCD	3.79	0.70	1.00			
2. Informal OSCD	3.77	0.87	0.87^**^	1.00		
3. Organizational commitment	5.34	1.24	0.69^**^	0.74^**^	1.00	
4. Turnover intention	2.21	0.77	-0.29^**^	-0.31^**^	-0.41^**^	1.00

^**^p < 0.01 (2-tailed)

### Hypothesis testing

The regression analysis results of the simple mediation models are summarised in Table 3. As can be seen, the results showed that both formal and informal OSCD had no significant direct relationship with turnover intentions (*β* = 0.03, *p* > 0.05; *β* = 0.02, *p* > 0.05), while having a significant positive direct relationship with organisational commitment (*β* = 0.65, *p* < 0.01; *β* = 0.69, *p* < 0.01). Simultaneously, organisational commitment had a significant negative direct relationship with turnover intentions (*β* = -0.36, *p* < 0.01; *β* = -0.35, *p* < 0.01). Based on these analysis results, H1 was rejected, H2 and H3 were supported.

**Table 3:** Regression analysis results of simple mediation models in PROCESS

Variables	Organizational commitment			Turnover intention			Organizational commitment			Turnover intention		
	*β*	*S.E*	*t*	*β*	*S.E*	*t*	*β*	*S.E*	*t*	*β*	*S.E*	*t*
Gender (female = 0)	-0.03	0.10	-0.63	-0.13^*^	0.08	-2.41	-0.04	0.10	-0.97	-0.13^*^	0.08	-2.40
Age	0.04	0.12	0.64	-0.18^*^	0.09	-2.05	0.05	0.11	0.75	-0.18^*^	0.09	-2.05
Marital status (unmarried = 0)	0.11^*^	0.17	2.18	-0.03	0.13	-0.48	0.07	0.16	1.51	-0.03	0.13	-0.47
Education	-0.07	0.07	-1.50	0.13^*^	0.06	2.32	-0.08	0.07	-1.91	0.13^*^	0.06	2.30
Professional title	-0.04	0.07	-0.66	-0.08	0.06	-1.13	-0.02	0.07	-0.31	-0.07	0.06	-1.10
Organizational tenure	0.05	0.08	0.64	0.14	0.06	1.51	0.02	0.08	0.24	0.13	0.06	1.49
Formal OSCD	0.65^**^	0.08	14.50	0.03	0.08	0.34						
organizational commitment				-0.36^**^	0.05	-4.83				-0.35^**^	0.05	-4.48
Informal OSCD							0.69^**^	0.06	16.52	0.02	0.07	0.19
Model summary	*R*^*2*^ = 0.51; *F* = 43.30; *p* < 0.001			*R*^*2*^ = 0.23; *F* = 10.50; *p* < 0.001			*R*^*2*^ = 0.57; *F* = 53.89; *p* < 0.001			*R*^*2*^ = 0.22; *F* = 10.48; *p* < 0.001		

^*^p < 0.05; ^**^p < 0.01; *β* is standardized coefficient, *S.E.* is standard error

The mediation effect of organisational commitment between OSCD and the turnover intentions of healthcare workers is shown in Table 4. The results showed that the total effects of formal and informal OSCD on healthcare workers’ turnover intentions were significant and negative (*B* = -0.23, 95%*CI* [-0.35, -0.10]; *B* = -0.20, 95%*CI* [-0.30, -0.10]). The direct effects of formal and informal OSCD on healthcare workers’ turnover intentions were insignificant (*B* = 0.03, 95%*CI* [-0.13, 0.19]; *B* = 0.01, 95%*CI* [-0.12, 0.15]). The indirect effects of formal and informal OSCD on healthcare workers’ turnover intentions were significant and negative (*B* = -0.25, 95%*CI* [-0.39, -0.15]; *B* = -0.22, 95%*CI* [-0.38, -0.12]). Therefore, organisational commitment played a fully mediating role in the relationship between OSCD and turnover intentions among healthcare workers. Thus, H4 was supported.

**Table 4:** Mediation effect of organizational commitment between OSCD and turnover intention

Dependent variable	Independent variable	Significance tests for effects				
			*B*	*S.E*	*Boot LLCI*	*Boot ULCI*
Turnover intention	Formal OSCD	Total	-0.23	0.06	-0.35	-0.10
		Direct	0.03	0.08	-0.13	0.19
		indirect	-0.25	0.06	-0.39	-0.15
Turnover intention	Informal OSCD	Total	-0.20	0.05	-0.30	-0.10
		Direct	0.01	0.07	-0.12	0.15
		indirect	-0.22	0.06	-0.38	-0.12

*B* is unstandardized effect value, *LLCI* is the lower level of the 95% confidence interval, *ULCI* is the upper level of the 95% confidence interval

## Discussion

Turnover of healthcare workers is a common problem in township hospitals in China. Reducing the turnover of healthcare workers is a key task for governments and organisational managers. This study investigated the turnover intentions of healthcare workers in township hospitals in Henan, China. After the controlling for socio-demographic variables including gender, age, marital status, education, professional title and organisational tenure, this study tested the relationship between OSCD, organisational commitment, and turnover intentions of healthcare workers. The results showed that three of the four hypotheses were supported and one was rejected.

This study measured the turnover intentions of healthcare workers in township hospitals. The turnover intention score (2.21) was lower than the median value (3), and also lower than that the score reported by Ma et al. in their study of the factors of turnover intentions among rural primary healthcare workers [25]. This result may be due to the continuous improvement in talent attraction and retention policies in rural primary health institutions [26]. Furthermore, one-way ANOVA revealed that there were individual differences in turnover intentions. First, female healthcare workers had stronger turnover intentions than males. This result is inconsistent with previous findings [5, 27]. An explanation for this may be that in the traditional gender-role ideology, men tend to play the breadwinner role, while women focus on family. Therefore, stable jobs are more important for men than women [28]. Second, healthcare workers, who were younger, unmarried, and highly educated with no professional title or work in the current institution for a short period of time, have higher turnover intentions. These findings are consistent with those of previous studies [29, 30].

The results of the study indicated a significant positive association between OSCD (formal and informal) and organisational commitment. Similar results have been reported in previous studies [31, 32]. For many people, their careers have important meanings in their lives. Therefore, when healthcare workers obtain career development assistance from their organisation, they feel that the organisation values their personal development and enhances organisational commitment [16]. Furthermore, the results indicated that organisational commitment was negatively related to healthcare workers’ turnover intentions. Psychologically, this was probably because healthcare workers who felt a sense of belonging and responsibility were more likely to stay in their current organisations. This finding is in line with those of previous studies [13, 33].

Interestingly, the results showed that OSCD (formal and informal) had a significant negative total effect on healthcare workers’ turnover intentions, which mainly came from its indirect effects. A direct relationship between OSCD (formal and informal) and turnover intentions was not found. In other words, organisational commitment played a fully mediating role in the relationship between turnover intentions among healthcare workers and OSCD (formal and informal). This finding is inconsistent with the view of some scholars who believe that OSCD has a direct negative relationship with turnover intentions [11]. These differences in results may be related to occupational backgrounds. As professionals, healthcare workers pay more attention to the role of OSCD in improving career development abilities and opportunities. Therefore, the OSCD is only a remote predictor of turnover intention and does not have a direct relationship with it.

### Implications

In this ‘new career’ era that emphasises the role of the individual, our study revealed a relationship between OSCD, organisational commitment, and turnover intentions. This study has important implications for talent retention in township hospitals.

A major contribution is its suggestion that policymakers and organisation managers should promote OSCD practices to improve healthcare workers’ organisational commitment and lower their turnover intentions. First, organisational managers must offer ability-fit jobs and more training and learning opportunities for healthcare workers, while formulating a reasonable performance appraisal system. Second, managers should pay attention to the important role of informal career development support measures such as mentoring, professional teams, and interest groups in improving organisational commitment and diluting turnover tendencies. Organisational managers can promote the development of these forms of informal support through appropriate incentives. Third, due to the limited strength of township hospitals, especially those located in remote areas, policymakers should constantly improve the relevant policies (financial support policy, continuing education policy, etc.) to assist township hospitals in supporting the career development of healthcare workers.

Another major insight is the importance of improving organisational commitment. When healthcare workers have a strong sense of belonging and responsibility toward their organisation, they are more willing to stay. Thus, hospital managers should adhere to ‘humanistic management’ and pay attention to humanistic care for healthcare workers.

Additionally, considering the differences in turnover intentions among different groups, policymakers and healthcare facility managers should adjust their talent retention policies and measures based on differences in socio-demographic characteristics.

### Limitations

The study has some limitations. First, the ability to infer the causal relationship between OSCD, organisational commitment, and turnover intentions was limited because this was a cross-sectional study. Second, we failed to identify and control all potential predictors of healthcare workers’ turnover intentions. Third, differences in the relationship between OSCD, organizational commitment, and turnover intention among different groups have not been explored. Fourth, the data analysis in this study was based on respondents’ answers, which may be inevitably biased. Finally, samples were obtained from a province in China, so the findings’ generalizability may be limited.

## Conclusion

This study analysed the relationship between OSCD, organisational commitment, and turnover intentions among healthcare workers in township hospitals in Henan, China. Our study suggests that there is a positive relationship between OSCD and organisational commitment and a negative relationship between organisational commitment and turnover intentions. Organisational commitment plays a fully mediating role between OSCD and turnover intentions. Furthermore, gender, age, marital status, education, professional title, and organisational tenure influence the turnover intentions of healthcare workers. These findings provide empirical evidence for government health policymakers and institutional human resource managers to formulate strategies for the retention of healthcare workers.

## Supplementary Information

**Additional file 1: **Descriptive statistics of participants in relation to OSCD and organizational commitment.

## Data Availability

The data that support the findings of this study are available from Healthy Central Plains Research Institute of Xinxiang Medical University. The data are available from the corresponding author upon reasonable request and with permission of Healthy Central Plains Research Institute of Xinxiang Medical University.

## Abbreviations

OSCD: Organizational support for career development
ANOVA: Analysis of Variance
SD: Standard deviation
S.E.: Standard error
LLCI: The lower level of the 95% confidence interval
ULCI: The upper level of the 95% confidence interval
