# Supplementary material for: The relationship between organisational support for career development, organisational commitment, and turnover intentions among healthcare workers in township hospitals of Henan, China
Source: BMC Prim Care. 2022 Jun 2;23:136. doi: 10.1186/s12875-022-01753-4 (PMC9161467; doi:10.1186/s12875-022-01753-4)
Supplement: Supplementary file 1 — Additional file 1: Descriptive statistics of participants in relation to OSCD and organizational commitment. [file 12875_2022_1753_MOESM1_ESM.docx]

**Descriptive statistics of participants in relation to OSCD and organizational commitment**

AS can be seen from Supplementary Table 1, healthcare workers’ organizational commitment had significant difference in age, marital status, education, professional title and organizational tenure. Healthcare workers’ perceived formal OSCD had significant difference in age, marital status, education, profession and profession title. Healthcare workers’ perceived informal OSCD had significant difference in age, marital status, education, profession title and organizational tenure.

**Supplementary Table** 1. Descriptive statistics of participants in relation to OSCD and organizational commitment (*N*=298)

| Characteristics | *N* (*%*) | Organizational commitment(*Mean*±*SD*) | *p* | Formal OSCD(*Mean*±*SD*) | *p* | Informal OSCD(*Mean*±*SD*) | *p* |
| --- | --- | --- | --- | --- | --- | --- | --- |
| Gender |  |  |  |  |  |  |  |
| Male | 130(43.62) | 5.34±1.41 | 0.94 | 3.82±0.77 | 0.65 | 3.81±0.92 | 0.46 |
| Female | 168(56.38) | 5.35±1.10 |  | 3.78±0.65 |  | 3.73±0.84 |  |
| Age |  |  |  |  |  |  |  |
| <30 | 72(24.16) | 4.92±1.58 | <0.01 | 3.60±0.82 | 0.02 | 3.51±1.05 | 0.01 |
| 30~ | 140(46.98) | 5.33±1.12 |  | 3.84±0.67 |  | 3.80±0.85 |  |
| 40~ | 86(28.86) | 5.71±1.00 |  | 3.88±0.61 |  | 3.92±0.69 |  |
| Marital status |  |  |  |  |  |  |  |
| Unmarried | 54(18.12) | 4.43±1.72 | <0.01 | 3.37±0.88 | <0.01 | 3.17±1.10 | <0.01 |
| Married | 244(81.88) | 5.54±1.01 |  | 3.89±0.62 |  | 3.90±0.76 |  |
| Education |  |  |  |  |  |  |  |
| High school/Technical school or below | 72(24.16) | 5.64±1.12 | <0.01 | 3.89±0.74 | 0.01 | 3.92±0.88 | 0.03 |
| Junior college | 120(40.27) | 5.49±1.13 |  | 3.88±0.59 |  | 3.83±0.73 |  |
| Bachelor or higher | 106(35.57) | 4.97±1.36 |  | 3.63±0.76 |  | 3.59±0.99 |  |
| Profession |  |  |  |  |  |  |  |
| Physicians | 152(51.01) | 5.19±1.33 | 0.14 | 3.72±0.68 | <0.01 | 3.72±0.84 | 0.18 |
| Nurses | 58(19.46) | 5.41±0.91 |  | 3.71±0.67 |  | 3.64±0.88 |  |
| Pharmacists | 18(6.04) | 5.89±0.71 |  | 4.33±0.49 |  | 4.17±0.78 |  |
| Medical laboratory workers | 16(5.37) | 5.28±1.81 |  | 4.02±0.83 |  | 3.78±1.11 |  |
| Other healthcare workers | 54(18.12) | 5.51±1.20 |  | 3.83±0.75 |  | 3.88±0.89 |  |
| Professional title |  |  |  |  |  |  |  |
| No | 60(20.13) | 5.03±1.64 | 0.01 | 3.56±0.79 | <0.01 | 3.50±1.02 | <0.01 |
| Junior | 114(38.26) | 5.25±1.08 |  | 3.82±0.53 |  | 3.77±0.72 |  |
| Middle | 92(30.87) | 5.47±1.13 |  | 3.79±0.76 |  | 3.76±0.95 |  |
| Senior | 32(10.74) | 5.86±1.09 |  | 4.18±0.77 |  | 4.27±0.67 |  |
| Organizational tenure |  |  |  |  |  |  |  |
| <5 | 80(26.85) | 5.14±1.59 | 0.01 | 3.74±0.83 | 0.47 | 3.73±1.00 | 0.05 |
| ~9 | 68(22.82) | 5.21±1.25 |  | 3.82±0.64 |  | 3.68±0.90 |  |
| ~19 | 90(30.20) | 5.30±0.99 |  | 3.74±0.69 |  | 3.68±0.84 |  |
| 20~ | 60(20.13) | 5.81±0.92 |  | 3.91±0.61 |  | 4.04±0.65 |  |
| Average monthly income (RMB Yuan) |  |  |  |  |  |  |  |
| ＜3000 | 124(41.61) | 5.40±1.31 | 0.75 | 3.82±0.70 | 0.82 | 3.83±0.87 | 0.44 |
| 3000~ | 102(34.23) | 5.32±1.18 |  | 3.78±0.58 |  | 3.68±0.77 |  |
| 4000~ | 72(24.16) | 5.26±1.22 |  | 3.76±0.86 |  | 3.77±1.02 |  |
| Hours worked per week |  |  |  |  |  |  |  |
| ≤40 | 68(22.82) | 5.50±1.25 | 0.46 | 3.82±0.73 | 0.80 | 3.82±0.87 | 0.56 |
| 41~56 | 142(47.65) | 5.32±1.22 |  | 3.81±0.70 |  | 3.79±0.89 |  |
| 57~ | 88(29.53) | 5.25±1.28 |  | 3.75±0.69 |  | 3.68±0.85 |  |
